# Point-Particle DNS and LES of Particle-Laden Turbulent flow - a state-of-the-art review

**DOI:** 10.1007/s10494-016-9765-y

**Published:** 2016-09-16

**Authors:** J. G. M. Kuerten

**Affiliations:** 1 Department of Mechanical Engineering, Eindhoven University of Technology, P.O. Box 513, 5600 MB Eindhoven, The Netherlands; 2 Faculty EEMCS, University of Twente, P.O. Box 217, 7500 AE Enschede, The Netherlands

**Keywords:** Particle-laden flow, DNS, LES

## Abstract

Particle-laden or droplet-laden turbulent flows occur in many industrial applications and in natural phenomena. Knowledge about the properties of these flows can help to improve the design of unit operations in industry and to predict for instance the occurrence of rain showers. This knowledge can be obtained from experimental research and from numerical simulations. In this paper a review is given of numerical simulation methods for particle-laden flows. There are various simulation methods possible. They range from methods in which all details, including the flow around each particle, are resolved, via point-particle methods, in which for each particle an equation of motion is solved, to Eulerian methods in which equations for particle concentration and velocity are solved. This review puts the emphasis on the intermediate class of methods, the Euler-Lagrange methods in which the continuous phase is described by an Eulerian approach and the dispersed phase in a Lagrangian way with equations of motion for each individual particle.

## Introduction: Overview of Simulation Methods

Particle-laden and droplet-laden turbulent flows are ubiquitous in nature and in industrial applications. Well-known examples are clouds, which consist of a large number of small water droplets, transport and sedimentation of sand particles in rivers and seas, separation of small particles from an air flow in industrial cyclones and evaporating milk droplets in spray dryers. Particle-laden turbulent flows are studied both experimentally and by means of numerical simulation. In this review only numerical simulation methods will be discussed. Many different numerical approaches exist, which can be applied depending on the application.

### Particle-resolved DNS

In the most detailed method the flow around each particle is resolved and the motion of a particle follows from the external forces and the hydrodynamic force exerted by the surrounding fluid. In case of droplets or bubbles, which can deform, the deformation also follows from the force exerted by the fluid. Since the flow around a particle needs to be resolved, this simulation method is only possible if the spacing of the computational grid is small compared to the size of a particle. This restricts the application of this method to particles that are large compared to the smallest scales of the turbulent flow and/or relatively small numbers of particles. Various numerical methods have been developed for particle-resolved direct numerical simulation.

In one of them a body-fitted spherical grid around the particles is used, sometimes embedded in a Cartesian grid for the whole computational domain. This method has been used to calculate the force on a single, fixed particle in decaying homogeneous isotropic turbulence [7, 24]. Very recently this method has been applied to analyze homogeneous isotropic turbulent flow around an array of 64 fixed spherical particles [111] with a size equal to twice the Kolmogorov length and a particle volume fraction of 0.001. Extension of this method to moving particles is not straightforward due to collisions that occur and lead to overlap of the spherical grid of one particle with another particle.

For bubbles moving in a turbulent flow front-tracking methods have been developed which allow the simulation of hundreds of bubbles [58, 105]. Larger numbers of particles in a turbulent flow have been reached by means of the immersed boundary method [107], where a Cartesian grid is used throughout the computational domain. There are various ways to approximate the boundary conditions on the surface of the particles, which does not coincide with grid lines. Picano et al. [78] recently applied this method to simulate 10,000 moving spherical particles in turbulent channel flow and studied the attenuation of turbulence caused by the particles. Also the lattice Boltmann method has been applied to the simulation of particle-laden turbulent flow [25]. This is another example where the grid is Cartesian and not aligned with the shape of the particles.

The last example mentioned here of a particle-resolved simulation method is Physalis [101, 121], in which a local analytical solution for the flow around each particle is used. This method has been applied to the simulation of hundreds of spherical particles in decaying homogeneous turbulence.

### Lagrangian point-particle methods

All of the above methods are restricted to rather small numbers of particles, which are not small compared to the Kolmogorov scale. A standard approach which can be used for simulations of millions of small particles is the point-particle approach. If the particle size is smaller than the Kolmogorov scale, the particle can be considered as a point particle and Lagrangian simulations, in which equations for each particle are solved, can be performed. This is the oldest type of Lagrangian simulation of particle-laden turbulent flow. An important quantity in this approach is the particle relaxation time, the time it takes a particle to adjust to the local, instantaneous flow. For very small values of the particle relaxation time, the particle velocity is in good approximation equal to the local, instantaneous fluid velocity. In this case particles behave as tracers. For larger values of the particle relaxation time, particles cannot follow the flow exactly and apart from a kinematic equation for the particle position, also an equation of motion for the particle velocity based on Newton’s second law of motion has to be solved.

Lagrangian methods can have different level of detail of description of the motion of the fluid. If, apart from the detailed flow around each particle, all scales of fluid motion up to the Kolmogorov scale are solved, we speak of point-particle direct numerical simulation (DNS). Just as for single-phase flow, also a filtered fluid velocity can be applied, leading to point-particle large-eddy simulation (LES). In that case the fluid velocity at the particle position is not exactly known, but only a filtered fluid velocity is available. In some applications the unresolved scales of the fluid velocity have a significant effect on the particle motion and, apart from a subgrid model in the equations governing the fluid, a subgrid model in the particle equations of motion is required.

Also, just as for single-phase flow, a statistical description of the fluid flow can be employed based on the Reynolds-averaged Navier-Stokes equation (RANS). This greatly reduces the computational efforts in case of statistically steady turbulent flows, since the mean flow quantities need to be calculated only once. Solution of the particle equations of motion can be done in a post-processing step. However, in such a simulation an additional model for the effects of the turbulence on the particles is required [73]. Without such a model the particles are not affected by turbulence and all particles starting at the same location would follow the same path. In order to take into account the effects of the turbulence on the particle motion, a stochastic turbulent dispersion model is required, which uses information from the RANS solution, for instance the Reynolds stress or the turbulence kinetic energy [23, 44]. Other types of stochastic dispersion models are based on a probability density function method [81, 102].

**Fig. 1:**
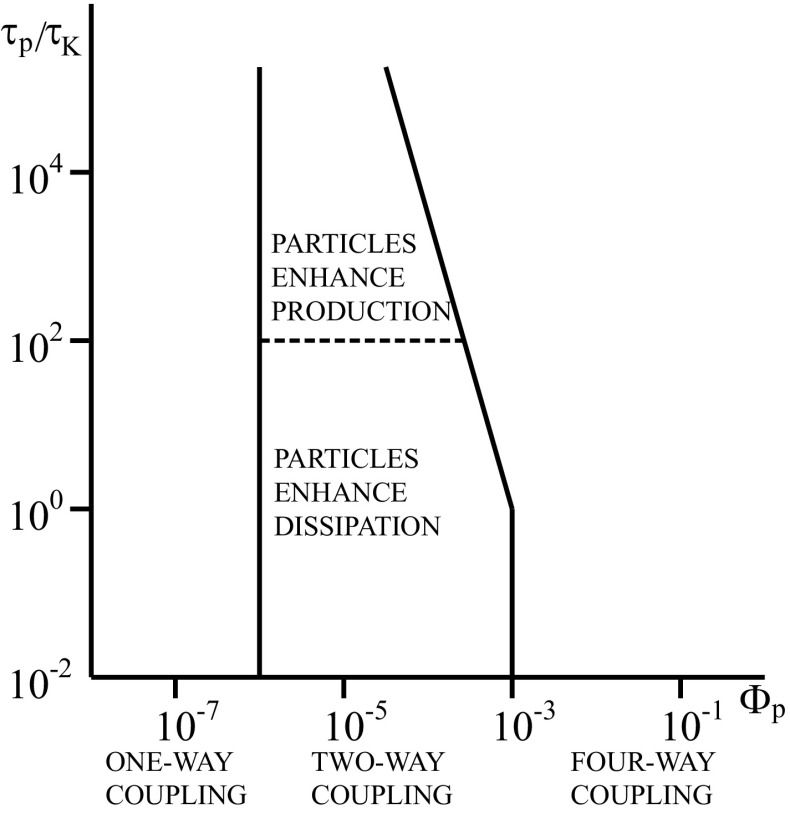
Different regimes in particle-laden flow. The particle volume fraction on the horizontal axis is denoted by Φ_*p*_. The vertical axis represents the ratio of the particle relaxation time *τ*_*p*_ to the Kolmogorov time *τ*_*K*_. This figure is taken after Elghobashi [34]

Lagrangian methods can also be distinguished according to the level of coupling between particles and fluid. If the particle volume fraction is sufficiently low, the particles do not influence the flow. This is the so-called one-way coupling regime. For higher particle volume fractions, the presence of the particles changes the properties of the flow and two-way coupling needs to be taken into account. For even higher volume fractions collisions between particles influence the simulation results. This is sometimes named four-way coupling. For homogeneous isotropic turbulence the limits of validity of the one-way and two-way coupling regimes have been suggested by Elghobashi [34], as shown in Fig. 1. In principle, all levels of coupling between particles and fluid can be applied to the three different levels of detail of the description of the turbulence, DNS, LES and RANS.

In several applications more physical phenomena play a role, such as heat exchange between the continuous and dispersed phase and mass transfer between the two phases by evaporation of droplets and condensation of vapor or by chemical reactions. Such extensions require additional equations, for instance for particle temperature and droplet mass.

### Eulerian methods

The Lagrangian point-particle approach has also been applied to particles that are not small compared to the Kolmogorov scale, in spite of its questionable validity in this case. Theoretically, the only possible approach for simulations with a large number of larger particles, or even larger numbers of small particles, is an Euler-Euler approach. In this approach particles are not described individually, but by concentration and velocity fields. Apart from the equations for the fluid phase, also partial differential equations for particle concentration and velocity have to be solved. The interaction between the particles and the fluid needs to be taken into account in the two momentum equations [28, 38]. If particles of different sizes, which interact differently with the flow, are present, a set of equations for each size class needs to be solved. The Euler-Euler approach is often used in the simulation of bubble columns and fluidized beds.

Eulerian methods have been developed for both direct numerical simulation and large-eddy simulation. An Euler-Euler LES method has been proposed by Moreau et al. [74], where the algebraic-closure-based moment method has been used to derive the LES model. Euler-Euler DNS has recently been applied by Masi et al. [67], who used a similar idea to make Euler-Euler DNS suitable for turbulence with mean shear and particles with larger Stokes numbers. The algebraic-closure-based moment method is based on the mesoscopic Eulerian formalism [38], in which the particle velocity field is partitioned in two contributions. One contribution is the mesoscopic Eulerian particle velocity field and the second represents the random uncorrelated motion of the particles. Modeling this second contribution is crucial for an effective Eulerian model, especially in the dilute regime.

In the following sections of this review paper the attention will be restricted to point-particle methods, in particular to point-particle DNS in Section 2 and to point-particle LES in Section 3. A number of other review papers have been published on numerical simulation of two-phase flow, which deal with other approaches. A few examples are the overview of both experimental and numerical approaches for turbulent dispersed multiphase flows by Balachandar and Eaton [8], the overview of particle-resolved DNS by Tenneti and Subramaniam [103] and the review about Lagrangian properties of particles in turbulent flow by Toschi and Bodenschatz [104]. The reviews about numerical simulation of gas-solid fluidized beds by Van der Hoef et al. [46] and by Deen et al. [30] describe a variety of the approaches mentioned above that can be applied to the various scales that play a role in this type of flow. A historical overview of much of the research on particle-laden flow, both experimental and numerical, with an emphasis on the research carried out in Stanford has been given by Eaton [32].

## DNS with Point-Particle Approach

### Particle equation of motion

The basis for almost all applications of point-particle DNS is the Maxey-Riley equation [68], which is the equation of motion of a spherical particle in a viscous fluid. Only in case passive particles are considered an equation of motion is not used. If **x**_*i*_ is the position of particle *i* and **v**_*i*_ its velocity, the kinematic equation reads

1 $$ \frac{d \, \textbf{x}_{i}(t)}{dt} = \textbf{v}_{i}. $$

For passive particles **v**_*i*_ = **u**(**x**_*i*_(*t*),*t*), where **u**(**x**,*t*) is the fluid velocity. Inertial particles satisfy equation of motion

2 $$ m_{i}\frac{d \, \textbf{v}_{i}(t)}{dt} = \sum\textbf{F}_{i}, $$

where *m*_*i*_ is the mass of particle *i* and the right-hand side consists of all forces acting on the particle.

The Maxey-Riley equation can be written as

3 $$ m_{i}\frac{d \, \textbf{v}_{i}(t)}{dt}=\textbf{F}_{B}+\textbf{F}_{PG}+\textbf{F}_{AM}+\textbf{F}_{D}+\textbf{F}_{hist}. $$

The terms on the right-hand side are respectively the buoyancy force, the force by the undisturbed velocity field, the added mass force, the drag force and the Basset history force. Maxey and Riley derived expressions for all these forces, which are valid in the limit of low Reynolds number, but they took into account the effects of finite particle size by the Faxén correction terms. Another requirement for the Maxey-Riley equation to be valid is that the particle is small compared to the Kolmogorov length. Details can be found in [68]. Apart from the forces given in (3), in several cases also the lift force is taken into account. Moreover, often correlations for the drag force valid for higher Reynolds numbers are used. Here only the more frequently used forms of the forces will be specified. Some authors considered the Faxén corrections for finite-sized particles. For example Homann and Bec [47] showed that the effect of these correction terms is significant for larger particles, but negligible if the particle diameter is equal to the Kolmogorov size or smaller.

The buoyancy force can be written as

4 $$ \textbf{F}_{B}=(\rho_{i}-\rho_{f})V_{i}\textbf{g} $$

with **g** the gravitational acceleration, *ρ*_*i*_ and *ρ*_*f*_ are the mass densities of particle *i* and of the fluid and *V*_*i*_ is the particle volume. The second term on the right-hand side of (3) is the force by the undisturbed velocity field,

5 $$ \textbf{F}_{PG}=m_{f,i}\frac{D\textbf{u}_{i}}{Dt}, $$

where

$$\frac{D}{Dt}=\frac{\partial}{\partial t}+\textbf{u}\cdot\nabla$$

is the material derivative, which is evaluated at the particle position. This force is often called pressure gradient force, since in an earlier derivation of the particle equation of motion, the viscous contribution to this term was neglected. Maxey and Riley [68], however, showed that both the pressure gradient and the viscous force should be included.

Without finite-size effects the added mass force can be written as

6 $$ \textbf{F}_{AM}=\frac12\rho_{f}V_{i}\left( \frac{D\textbf{u}_{i}}{Dt}-\frac{d\textbf{v}_{i}(t)}{dt}\right). $$

Note that the first term between the parentheses has the same shape as the pressure gradient force, while the second term has the same shape as the left-hand side of (3).

In most papers the drag force in (3) is taken equal to

7 $$ \textbf{F}_{D}=m_{i}\frac{(\textbf{u}(\textbf{x}_{i}(t),t)-\textbf{v}_{i})}{\tau_{i}}(1+0.15\Re_{i}^{0.687}).$$

In this equation *τ*_*i*_ is the particle relaxation time, given by

8 $$ \tau_{i}=\frac{\rho_{i} {d_{i}^{2}}}{18\mu} $$

and Re_*i*_ is the particle Reynolds number, given by

9 $$ \Re_{i}=\frac{\rho_{f} d_{i}|\textbf{u}(\textbf{x}_{i}(t),t)-\textbf{v}_{i}|}{\mu}, $$

where *μ* is the dynamic viscosity of the fluid and *d*_*i*_ is the particle diameter. In the expression for the drag force also finite size effects have been disregarded and the Schiller-Naumann correlation valid for particle Reynolds numbers between 0 and 1000 has been applied [27].

Finally, the last term in (3) is the history force, which can be written as

10 $$ \textbf{F}_{hist}=\frac32(\pi\rho_{f}\mu)^{1/2}{d_{i}^{2}}{{\int}_{0}^{t}}\frac{\frac{d}{d\tau}(\textbf{u}_{i}(\tau)- \textbf{v}_{i}(\tau))}{(t-\tau)^{1/2}}d\tau, $$

if again the effects of finite particle size are ignored.

In several studies also the lift force has been included. There are two mechanisms for lift force, *i.e.* a force perpendicular to the relative velocity between fluid and particle. One is caused by a shear in the fluid velocity, the shear-induced or Saffman lift force [6], and the other is caused by particle rotation, the slip-rotation lift force or Magnus force [86].

### Relevance of various forces

The relevance of the forces mentioned in the previous section in various applications has been discussed in a number of papers. In [4] it has been observed that for small and heavy particles, where heavy means that *ρ*_*i*_≫*ρ*_*f*_, the pressure gradient force, added mass force and Basset history force are small compared to the drag force. For light particles, in particular for bubbles in a liquid or for neutrally buoyant particles, these three forces become important. It has for example been shown that the history force cannot be neglected in case of neutrally buoyant particles [1] and that more forces need to be taken into account if *ρ*_*i*_/*ρ*_*f*_<100 [35]. Also the lift force is more relevant for light particles than for heavy particles. A second reason why the history force is often not taken into account is the high computational and memory costs for calculating the integral over the history of the particle. Recently, however, a method has been developed to overcome this problem [45].

In many applications, in particular for solid particles or droplets in a turbulent gas flow, the mass density ratio is so large that only the drag force and gravity force need to be taken into account. This approach has been followed in many papers for various flows.

### One-way coupling

To the best of my knowledge the oldest paper on point-particle DNS, is by Riley and Patterson and dates as far back as 1974 [85]. They inserted both passive particles and inertial spherical particles in decaying homogeneous, isotropic turbulence. Due to the modest computational resources at that time the Reynolds number was quite low, Re_*λ*_<35, and only 32^3^ grid points and 432 particles were used. The equation of motion for the inertial particles contained only the linear drag force. The paper focussed on Eulerian and Lagrangian temporal velocity autocorrelation functions. For short times the Lagrangian autocorrelation is larger than the Eulerian and the opposite is true for larger times. Moreover, the Lagrangian velocity correlation increases with increasing particle relaxation time.

Yeung and Pope [119] and Balanchandar and Maxey [9] studied the accuracy of various methods for the interpolation of the fluid velocity to the particle positions in DNS of forced and decaying homogeneous isotropic turbulence. They studied methods ranging from linear interpolation to high-order Lagrangian and Hermitian interpolation methods and interpolations based on splines. The accuracy of the interpolation is not only determined by the interpolation method, but also by the resolution of the DNS. In general third-order accurate interpolation is required to predict Lagrangian velocity statistics with sufficient accuracy [119].

McLaughlin [69] has probably been the first to perform one-way coupled point-particle DNS of turbulent channel flow. He considered this flow at a bulk Reynolds number based on half the channel height of 2000 and studied the deposition of aerosol particles. Because of the high mass density ratio he considered only the drag force and the Saffman lift force, which turned out to be important in the viscous sublayer and hence for the deposition and accumulation of particles. Bernard et al. [12] studied the motion of passive particles in DNS of turbulent channel flow in order to analyze the origin of the Reynolds stress.

Kontomaris et al. [49] studied the dispersion of passive particles in DNS of turbulent channel flow at a Reynolds number of 9048 based on twice the channel height and the bulk velocity. They applied a Fourier-Chebyshev pseudo-spectral method with a rather coarse resolution compared to later work. They focussed on the accuracy of various methods for the interpolation of the fluid velocity to the particle positions.

One-way coupled point-particle DNS has been reported in many papers, and for various kinds of flow, *e.g.*, channel flow [65], non-rotating [61, 79] and rotating pipe flow [36] and homogeneous isotropic turbulence, both forced [97, 98] and decaying [35]. Elghobashi and Truesdell [35] simulated inertial particles moving in decaying homogeneous isotropic turbulence with initially Re_*λ*_=25 and taking all terms in the particle equation of motion according to Maxey and Riley [68] into account. The mean-square particle displacement agreed well with measurement results, although the Reynolds number in the experiment was higher by a factor of three.

In particle-laden wall-bounded turbulent flows preferential concentration of particles plays an important role. In homogeneous isotropic turbulence preferential concentration results in non-uniform local particle concentration, but the average particle concentration in time is uniform. In inhomogeneous turbulent flows, particles tend to move to regions with lower turbulence kinetic energy. This is called turbophoresis [84] and results in larger particle concentrations near the walls in channel and pipe flow [64, 120]. In [65] results of a benchmark problem for particle-laden turbulent channel flow at a frictional Reynolds number of 150 are reported. The benchmark includes results of a number of research groups with their own numerical method. Although the differences between the results are in general small, in some quantities, especially in the enhanced mean particle concentration close to the wall, for which turbophoresis is the main mechanism, non-negligible differences have been observed.

### Two-way coupling

One-way coupling is only applicable in dilute flows, where the (local) particle volume fraction is below 10^−5^ [33, 34]. For larger particle volume fractions the effects of the particles on the turbulence can no longer be disregarded and the reaction force of the particles on the flow needs to be taken into account. This requires the solution of two problems. The first is that all expressions for the hydrodynamic forces on a particle mentioned above require the knowledge of the undisturbed fluid velocity at the position of the particle. If the effect of the particles on the fluid is taken into account, this undisturbed velocity is not directly known. Boivin et al. discussed this issue [16] and argued that the difference between the undisturbed and disturbed fluid velocity is small if the particle diameter is small compared to the grid size. Since in DNS the grid size is almost always larger than the Kolmogorov length and the particle diameter has to be small compared to the Kolmogorov length for the point-particle approach to be applicable, this condition is satisfied.

The second problem is that the two-way coupling term, *i.e.*, the force exerted by a particle on the fluid is localized at the position of the particle, and can thus be represented by a Dirac delta-function. Especially in case of a relatively small number of particles, this results in a rather erratic distribution of this force over the computational domain. Therefore, usually the two-way coupling term is distributed over a number of neighboring grid points by means of a projection operator or allocated to the grid cell in which the particle is located. The latter approach is for instance used in [97], which is an example of a particle-source-in-cell method. It has, however, been shown that a projection method results in a smaller loss of kinetic energy [16].

Eaton [32] also discussed several problems related to two-way coupling. His first issue is that particles are typically larger than the grid cell, so that they cannot be represented by a point. As already pointed out above, in almost all numerical simulation methods, the grid size is larger than the Kolmogorov length and if the particle is smaller than the Kolmogorov length, it is certainly smaller than the grid size. Therefore, this argument is most often not problematic. Eaton’s second objection against two-way coupling is that for dilute flows, the two-way coupling term is “spotty”, as most grid cells do not contain a particle. This certainly depends on the problem. Simulations [54, 87] have been carried out with many very small particles, which are so small that the flow is still dilute, for example with a particle volume fraction on the order of 10^−4^. In the simulations cited the number of particles is comparable to the number of grid cells. The non-smooth character of the two-way coupling force does not lead to problems in the numerical method. Moreover, statistical results of the simulations, averaged over a sufficiently long time, appear not to be affected by the non-smooth two-way coupling force.

Boivin et al. [16] studied the influence of the presence of particles at different mass loading on the turbulence kinetic energy and dissipation rate for homogeneous isotropic turbulence. They noticed that both quantities decrease with increasing mass loading and that this decrease depends on the particle size (Stokes number). By considering the energy spectra, they observed that the turbulence spectral density is attenuated by larger particles and increased by smaller particles.

Pan and Banerjee [77] performed two-way coupled point-particle DNS of turbulent channel flow. They did not take the added-mass force and Basset history force into account, but included the pressure gradient force. The particle volume fraction was close to 10^−4^. For the two-way coupling force on the fluid a Stokeslet was applied for each particle. They observed that particles smaller than the Kolmogorov length suppress the turbulence, whereas larger particles enhance turbulence.

Zhao et al. [123, 124] performed two-way coupled point-particle DNS in turbulent channel flow at a frictional Reynolds number, based on half the channel height, of 180. A striking result is that particles reduce the drag, resulting in a larger bulk fluid flow rate at the same frictional Reynolds number compared to the unladen flow. Just like [16] they observed that the fluid velocity fluctuations in the streamwise direction are enhanced by the presence of particles, whereas the other two components and the Reynolds stress are significantly decreased. They also studied the effect of particles on coherent structures and reported an increase of their size.

Lee and Lee [56] performed two-way coupled point-particle DNS of turbulent channel flow at Re_*τ*_=180 taking into account only the nonlinear drag force exerted by the fluid on the particles. They considered particles with various Stokes numbers, keeping the mass loading constant and focused on the effects of the particles on the turbulence. The main result is that particles of small Stokes number increase turbulence intensities, the Reynolds stress, the viscous dissipation and the fluid acceleration statistics, whereas particles of larger Stokes numbers suppress the turbulence intensities. They explained this result by studying the interaction of the particles with the sweeps and ejections close to the walls. Note, however, that the particle volume fraction in the case with the lowest Stokes number is so high (10^−3^), that the disregard of particle collisions is questionable.

In [52] apart from momentum exchange also heat exchange between particles and fluid has been taken into account in differentially heated turbulent channel flow at frictional Reynolds numbers of 150 and 395. It was observed that the presence of small heavy particles with a specific heat larger than that of the fluid, gives rise to enhanced heat transfer between the two walls of the channel. This is caused by a combination of turbophoresis, which drives the particles toward the walls of the channel and a difference in mean temperature between particles and fluid close to the walls. Another result of this research is that the incorporation of two-way coupling reduces the effect of turbophoresis, resulting in lower particle concentrations close to the walls compared to one-way coupled simulations. This can be explained by the reduction in the wall-normal fluid velocity fluctuations due to two-way coupling.

This research has been further extended to include mass transfer between the dispersed phase, which consisted of water droplets, and the carrier phase, a combination of dry air and water vapor. Russo et al. [87] considered an incompressible formulation of the carrier gas, whereas Bukhvostova et al. [21] included the effects of changes in the total mass density caused by evaporation of droplets and condensation of water vapor, by applying a compressible formulation of the carrier gas. The possibility of phase change results in an even higher enhancement of the heat transfer between the walls of the channel, caused by the enthalpy of evaporation which results in a larger difference in mean temperature between droplets and gas than without phase change. For mild initial conditions the results of the incompressible and compressible formulation do not show significant differences. However, a lower initial relative humidity, leading to fast initial evaporation of droplets, results in differences on the order of 15 % in mean thermal properties of the system [22]. A drawback of the compressible formulation is the severe restriction on the time step if an explicit time-marching method is applied, since realistic Mach numbers are very small. This can be avoided by a low-Mach-number algorithm, which has been shown to yield results in very good agreement with the fully compressible formulation [20].

In earlier research DNS of droplet-laden turbulent flow has been studied for the case of forced homogeneous isotropic turbulence [66]. In that work only evaporation and no condensation of vapor has been considered and the droplet equations were simplified in the sense that it was assumed that the heat capacities of the liquid and vapor are constant and equal. In this paper the influence of varying the initial droplet temperature, mass loading and size on the evaporation rate and on the PDF of the droplet diameter has been investigated.

Evaporating droplets have also been considered in DNS of a confined mixing layer with one stream of hydrocarbon droplets [71]. In this research also two-way coupling of mass, momentum and energy has been incorporated. It was observed that an increased mass loading results in attenuation of the growth rate of the mixing layer and of the kinetic energy. This research has later been extended to higher Reynolds numbers [72] in order to study the role of the subgrid scales on transport, heating and evaporation of droplets by means of an *a priori* analysis of the DNS results and in order to propose subgrid models.

Another example where mass exchange between the dispersed phase and the carrier gas is relevant, is pyrolysis of biomass particles in a turbulent gas flow, where during pyrolysis the particles release volatile gases. This process has been studied by means of point-particle DNS in [88]. In order to model the processes taking place inside each particle, such as the moving front between the virgin biomass and the char, which results after pyrolysis, and the temperature profile, for each particle an additional set of ordinary differential equations has been solved. These equations represent a simplified model of the partial differential equation modeling the conduction, convection and heat of reaction inside the particle. The paper studied the relevance of two-way coupling and it appeared that two-way coupling starts influencing the conversion time of the particles at particle volume fractions on the order of 10^−5^, which is close to the boundary between one-way and two-way coupling indicated in [34] for momentum transfer.

### Four-way coupling

For higher particle volume fractions the particles do not only influence the continuous phase, but also the interaction between particles becomes more and more important. According to the diagram by Elghobashi [34], particle collisions have an effect on the results if the particle volume fraction is larger than 10^−3^ for homogeneous, isotropic turbulence. Simulations in which particle collisions are taken into account are frequently called four-way coupled simulations. Particle collisions require two additional elements in the simulation: an algorithm that searches collisions and a method that determines the result of a collision. In general it is assumed that the particle volume fraction and the duration of a collision are sufficiently small that only binary collisions need to be considered. In this section we will only consider spherical particles. Collision algorithms for non-spherical particles have also been developed and studied [93, 122]. Zhao et al. [122] considered ellipsoidal particles and used an extension of a collision search algorithm for spherical particles by treating each ellipsoid as a set of overlapping fictitious spheres, whose hull approximately corresponds to the shape of the ellipsoid.

Collision search algorithms can be divided into deterministic and stochastic methods. The simplest deterministic collision search algorithm considers all particle pairs. However, the cost of this algorithm is proportional to the square of the number of particles and becomes prohibitively expensive in most cases. More efficient search algorithms have been developed that are based on nearest-neighbor lists or domain decomposition. Nearest-neighbor lists are also employed in molecular dynamics simulations. Since in a time step Δ*t* the distance between two particles cannot change more than by 2*v*Δ*t*, where *v* is the maximum magnitude of particle velocity, only particle pairs within this distance need to be considered. If *N*_*par*_ is the number of particles, this results in a maximum reduction of the computational cost of the search algorithm to order *N*_*par*_ log*N*_*par*_. Hoomans et al. [48] employed square neighbor lists to determine all collision pairs in simulations of two-dimensional gas-fluidized beds. Vreman [109] applied a similar algorithm, but based on a decomposition of the domain in small blocks, to study turbulent particle-laden pipe flow.

An alternative way to avoid the computationally expensive particle-pair-collision search algorithm is to use a stochastic collision model. Such a stochastic model has first been proposed and employed by Oesterle and Petitjean [75] for gas-solid flow in a horizontal pipe. In this method single particles are tracked and in every time interval the fraction of particles that collide is determined from a collision probability. The collision probability involves the cross-sectional area of a particle pair, the relative velocity of two particles, the particle number density and the radial distribution function [100]. Due to preferential concentration of particles, the radial distribution function is not easy to determine. Moreover, the relative velocity of two colliding particles is strongly influenced by the correlation between two inertial particles at small distance, which makes this problem more complicated than in kinetic gas theory [114]. Stochastic particle-collision models have been further developed by *e.g.* Berlemont et al. [11] and Sommerfeld [96].

The second element of four-way coupling is the collision algorithm, which determines the outcome of a collision. Two basic types of models can be distinguished: hard-sphere and soft-sphere collision models. In the hard-sphere model two particles only feel each other the moment they touch and the collision occurs instantaneously. In the soft-sphere approach the relative motion of two colliding particles is governed by an equation of motion which involves the contact force between the two particles. For the contact force a linear spring-dash-pot model can be applied [106]. For very dense systems, the hard-sphere model cannot be applied, since it leads to very close packing and low relative velocities. This may ultimately result in overlapping particles. An advantage of the hard-sphere model is that the post-collision velocities can be calculated analytically from conservation of momentum and energy. Energy does not need to be conserved; inelastic collisions in which part of the kinetic energy of the relative motion of the two particles is transferred into heat can easily be incorporated as well.

In most simulations that have been performed with four-way coupling not only the linear momentum of the particles is taken into account, but also the particle rotation. During a collision both the particle linear and angular momentum are exchanged, in such a way that the sums of the linear and the angular momentum of the two collision partners are conserved [59]. Inelasticity of collisions can be incorporated by the coefficient of normal restitution, which reduces the normal component of the relative velocity during a collision. If the tangential component of the relative velocity is smaller than a certain threshold value, the particles undergo a sticking collision, otherwise a sliding collision [59].

Four-way coupled DNS of turbulent channel and pipe flow have been performed by a number of researchers. Vreman [109] studied the effects of small, heavy particles in vertical pipe flow of air for a wide range of mass loadings between 0.1 and 30. He incorporated a model for wall roughness and concluded that the effects of this model are much larger than effects of changes in the particle model, such as incorporation of a lift force and variations in collision parameters. Wall roughness turned out to be necessary to obtain agreement with experimental results. The turbulence intensities of the air reduce strongly with increasing particle mass loading.

Similar results have been obtained for particle-laden turbulent channel flow at mass loadings between 0.2 and 2 [31, 57]. In this flow the particles inhibit the transfer of energy from the streamwise to the spanwise and wall-normal velocity components, which increases the anisotropy of the turbulence. It has also been observed that particle collisions result in a significant reduction of turbophoresis. This latter observation has been studied in more detail for droplet-laden turbulent channel flow [54], where it appeared that even at a relatively low overall droplet volume fraction of 10^−4^, droplet collisions have a large impact on the droplet concentration profile. The combination of turbophoresis and preferential concentration results in local droplet concentrations which are much larger than the average volume fraction. The effect of collisions on the heat transfer between the walls of the channels is, however, not as big as on the droplet concentration. This can be explained by the higher difference in mean droplet and gas temperature as compared to two-way coupled simulations [87], resulting from the lower heat exchange area between gas and droplets in the near-wall region.

Vreman [110] performed four-way coupled point-particle DNS of downward gas-solid flow in a vertical channel at Re_*τ*_=642 and with a mass loading of 0.8. He considered smooth and rough walls, where rough walls were modeled by fixing tiny spherical particles on the walls. Rough walls enhance the turbulence attenuation caused by the free solid particles. Moreover, he decomposed the force exerted by the particles on the gas into three contributions: the spatial average of the mean force, the non-uniform part of the mean force and the fluctuating part of the force. He showed that the second contribution, the non-uniform part of the mean feedback force, has a significant contribution to the turbulence attenuation caused by the particles.

Some papers studied the particle collision frequency, which is an important quantity in stochastic collision algorithms. Since nearby inertial particles are correlated, the collision frequency differs from the theoretical expression that can be derived for molecules from kinetic gas theory. Sundaram and Collins [100] derived an estimate for the collision frequency, *i.e*, the number of collisions per unit volume and time, for inertial mono-disperse particles in a turbulent flow based on the cylindrical volume swept by a single particle per unit time. This estimate depends on the particle number density, on the relative velocity of two particles just before a collision and on the particle radial distribution function at contact. A second estimate of the collision frequency is the so-called spherical formulation by Wang et al. [113, 114], According to Saffman and Turner [89], the collision frequency in turbulent flows can be based on the net inward flux into a sphere of radius *d*_*p*_ around a particle.

Sundaram and Collins [100] calculated the particle collision frequency in simulations of particle-laden homogeneous isotropic turbulence for particles of various Stokes numbers. In their simulations the particle volume fraction is so low that they ignored the effect of the particles on the flow, but they carried out the collisions. For very small Stokes numbers the particles behave in agreement with the prediction of Saffman and Turner [89], while for very large Stokes numbers the particle collisions are more in agreement with kinetic theory. In the intermediate range a more complex behavior is observed, which is a consequence of preferential concentration and reduced correlation between neighboring particles. Both phenomena result in increased collision rates. In [83] the dependence of the radial distribution function on the turbulence and particle parameters was studied by means of DNS of particle-laden homogeneous isotropic turbulence.

Wang et al. [114] studied a similar system, but they only calculated the collision frequency without implementing the post-collision particle positions and velocities. In this way particles are allowed to overlap. In this way they avoided artificial repeated collisions, which may occur if particles collide with low relative velocity and the surrounding fluid field pushes the particles toward each other after a collision. They determined the collision frequency, the relative velocity of two colliding particles and the radial distribution function at contact for particles of various Stokes numbers and studied the effects of preferential particle concentration and relative velocity on the collision frequency separately. They concluded that the spherical formulation of the collision frequency is more accurate than the cylindrical formulation, if the particle concentration has reached a stationary state.

**Fig. 2:**
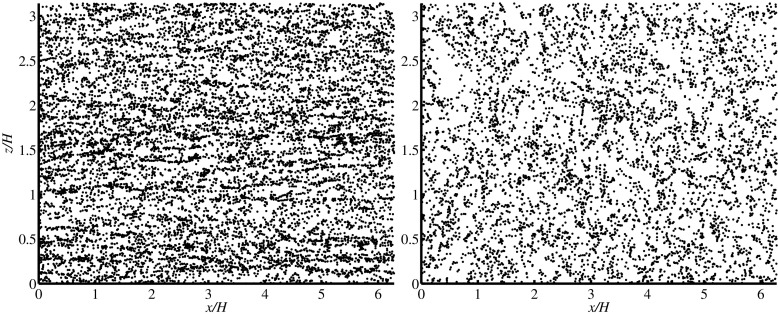
Instantaneous particle positions for point-particle DNS of turbulent channel flow at Re_*τ*_=950, St=10 and an overall particle volume fraction of 9×10^−5^. The figures show a small slice parallel to the walls for 7.45<*y* ^+^<7.55 (left) and for 949.85<*y* ^+^<950.15 (right), and *y* ^+^ is the wall-normal coordinate in wall units

Collision frequencies have been determined for inhomogeneous turbulent flow in [54] for the case of droplet-laden turbulent channel flow at a frictional Reynolds number of 150. In inhomogeneous turbulent flows the particle number density, mean relative velocity and radial distribution function are all dependent on the coordinate in which the turbulence is homogeneous, in this case the wall-normal coordinate. Due to turbophoresis, the particle number density close to the walls is larger than in the center of the channel. The effect of this on the collision frequency is enhanced by the larger mean relative velocity near the walls. In turbulent channel flow preferential concentration not only results in larger particle concentrations near the walls. Both near the walls and in the center of the channel local particle clustering also plays an important role. Near the walls the particles are clustered in the low-speed streaks. In the center of the channels regions with large particle concentration and regions void of particles can be observed. Both structures have a quite long life time. Typical examples of both regions are shown in Fig. 2 for particle-laden turbulent channel flow at Re_*τ*_=950 with particles of St=10 and an overall particle volume fraction of 9×10^−5^. These results have been determined by point-particle DNS.

**Fig. 3:**
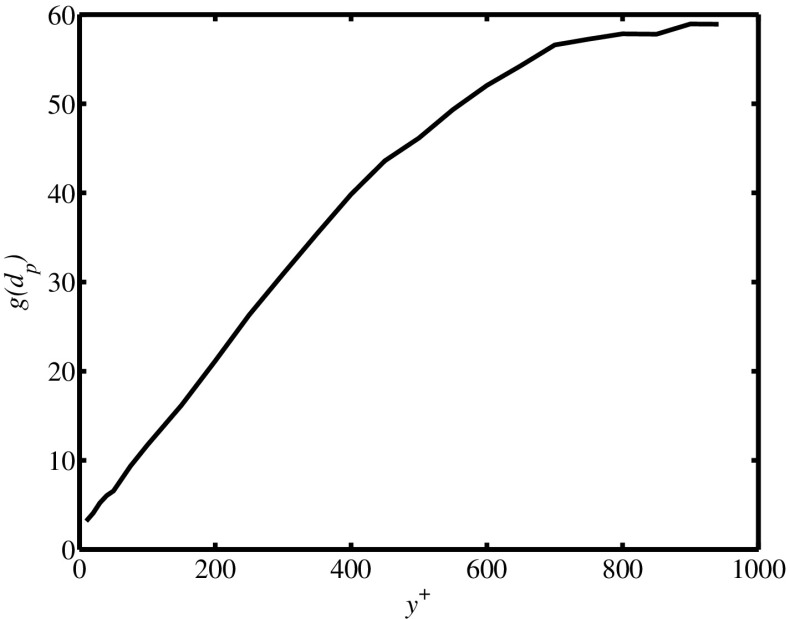
Radial distribution function at contact as a function of the wall-normal coordinate in wall units determined from point-particle DNS of turbulent channel flow at Re_*τ*_=950, St=10 and an overall particle volume fraction of 9×10^−5^

The radial distribution function at contact resulting from this DNS is shown in Fig. 3 as a function of the wall-normal coordinate.

## LES with Point-Particle Approach

### Subgrid modeling in the particle equation of motion

Instead of DNS also LES can be used for the description of the continuous phase in point-particle simulations. This implies that filtered fluid quantities are employed, usually defined by

11 $$ \bar{u}({\textbf{x}},t)={\int}_{V}G({\textbf{x}}-{\textbf{y}})u({\textbf{y}},t)d\textbf{y}, $$

where *G* is a convolution kernel and *u* a velocity component or the pressure. Possible filter functions are a top-hat filter, which is only unequal to zero inside a small rectangular domain or a Gaussian filter. The governing equations for the filtered fluid velocity are derived by applying the filter operation to the Navier-Stokes equation and to the continuity equation in case of incompressible flow [40]. Due to the non-linearities in the Navier-Stokes equation some terms appear which cannot be expressed in filtered quantities only, but also contain unfiltered quantities, for example a product of two unfiltered velocity components. Such terms have to be closed by a model, which expresses them as functions of the filtered quantities.

The most important term that needs to be closed is the subgrid stress tensor, which is defined by

12 $$ \tau_{ij}=\overline{u_{i}u_{j}}-\bar{u}_{i}\bar{u}_{j} $$

for incompressible flow, where *u*_*i*_ is a component of the fluid velocity. Various subgrid models have been proposed. The best known is the Smagorinsky model [95], which is based on the eddy-viscosity assumption, which renders the model dissipative. This model has later been improved by including corrections near a wall and by the dynamic procedure, which reduces the eddy viscosity in laminar regions [39]. Other subgrid models are based on the scale-similarity assumption [10], a Taylor series expansion [26], or approximate deconvolution [99]. For many types of turbulent flow LES has become a valuable simulation method, because of the significantly reduced computational costs as compared to DNS.

If LES is applied to the continuous phase in point-particle simulations of particle-laden turbulent flow, also the particle equations of motion contain terms that need to be closed. In particular, the pressure gradient force (5), the added-mass force (6), the drag force (7) and the history force (10) contain the unfiltered fluid velocity, which is unknown. In cases where the particle relaxation time is large compared to the typical time scales of the turbulent flow and to the smallest time scale resolved in the LES, the subgrid scales in the fluid velocity do not influence the particle motion significantly. In such cases a subgrid model in the particle equation of motion is not required and this approach has been followed in several papers [108, 115, 118]. The *a priori* and *a posteriori* study of Armenio et al. [5] showed that the subgrid scales have only a small effect on the particle motion, but they restricted this study to quantities and test cases where this is typically the case. It has later been shown [53] that disregard of subgrid scales has a large effect on turbophoresis in LES of particle-laden turbulent channel flow, if the particle relaxation time is of the same order of magnitude as the Kolmogorov time.

Two different types of models have been developed to account for the missing subgrid scales in the particle equation of motion. In the first type a stochastic model is applied, either by adding broadband stochastic noise forcing to the Navier-Stokes equation for the continuous phase [50], or by adding an additional velocity to the particle equation of motion [94]. The second type uses approximate deconvolution of the filtered fluid velocity to approximate the unfiltered fluid velocity, which is then used in the particle equation of motion [51].

Stochastic models for the subgrid contributions to the fluid velocity, which can be used as a subgrid model in the particle equation of motion, are often based on models that have originally been developed for the Reynolds-averaged Navier-Stokes equation. Some of them use a generalized Langevin model of the fluid velocity along the path of a particle and applied this to homogeneous isotropic turbulence [94], while others use a transport equation for the subgrid scale kinetic energy in particle-laden turbulent channel flow [116]. Shotorban and Mashayek [91] performed *a priori* and *a posteriori* tests of particle-laden decaying homogeneous isotropic turbulence with a stochastic model for the subgrid contributions to the fluid velocity. The *a priori* tests showed a very good agreement in the particle kinetic energy for lower Stokes numbers, whereas the use of the subgrid model appeared less necessary for particles with larger Stokes numbers. These results were confirmed by the *a posteriori* simulations.

In [3] three different stochastic models were considered in LES of particle-laden channel flow. The first is based on a transport equation for the subgrid scale kinetic energy, while the other two include the effects of temporal correlation in the subgrid scale contributions to the fluid velocity and the effects of anisotropy. It was shown that both additional effects are especially important in the near-wall region. The RMS of the particle velocity in wall-normal and spanwise direction is strongly influenced by the subgrid contributions to the fluid velocity.

Fede and Simonin [37] studied the effects of the subgrid scales on particle motion in forced homogeneous isotropic turbulence. They both performed DNS to study the small-scale fluid velocity fluctuations seen by the particle, and solved the particle equation of motion with explicitly filtered fluid velocity fields to evaluate the effect of the subgrid scales on particle statistics. Not surprisingly, they found that particle dispersion and kinetic energy are only affected by the filtering if a significant part of the turbulence kinetic energy is removed by the filter. However, particle accumulation and collision rates are significantly influenced when the particle relaxation time is of the same order or smaller than the subgrid Lagrangian integral time scale measured along particle paths.

Bini and Jones [14] focused on a stochastic model for the subgrid contributions to the fluid velocity that is able to reproduce the far from Gaussian behavior of the particle acceleration observed in experiments and direct numerical simulation. They achieved this through a nonlinear stochastic differential equation for the relative velocity between particle and fluid. In a later paper they applied this idea to a droplet-laden spatially developing mixing layer [15].

Pozorski and Apte [80] investigated the effect of using the filtered velocity field in the particle equation of motion on the particle motion in homogeneous isotropic turbulence. They especially considered and quantified the changes in preferential concentration patterns of particles. They proposed a stochastic Langevin model for the subgrid contributions to the fluid velocity based on the subgrid scale turbulence kinetic energy, which results in a correct reconstruction of the particle turbulence kinetic energy.

The first paper on approximate deconvolution as a subgrid model [53] considered LES of particle-laden turbulent channel flow at Re_*τ*_=150 and studied in particular the question whether LES is capable of predicting turbophoresis. They used the dynamic eddy-viscosity model [39] to describe the fluid flow and inverted the (implicitly defined) filter in Fourier space in the two periodic directions and numerically in the wall-normal direction. Approximate deconvolution (ADM) is only able to recover the energy in the resolved scales. The scales beyond the cut-off wave length cannot be retrieved in this way. Especially in the region of intermediate Stokes numbers, when the particle relaxation time is of the same order of magnitude as the Kolomogorov time, application of the filtered fluid velocity in the particle equation of motion results in significantly under-predicted turbophoresis. The subgrid model based on approximate deconvolution gives a significant improvement. This research was later extended to a higher Reynolds number [51] and by considering also the approximate deconvolution model [99] as a subgrid model in the fluid equation.

Shotorban and Mashayek [90] developed a similar idea and applied this to LES of homogeneous shear flow where the dynamic eddy-viscosity model [39] was applied to the fluid equation. They studied the effect of the subgrid model on the particle turbulence kinetic energy and turbulence diffusivity and also found a substantial improvement compared to simulations without subgrid model in the particle equation of motion. In a later paper [92] the same authors also obtained a beneficial effect of the subgrid model in the prediction of preferential concentration of particles in homogeneous shear flow.

Marchioli et al. [62] studied the effects of ADM in particle-laden channel flow at Re_*τ*_=150 for particles at several Stokes numbers and for coarse and fine LES. Only in the fine LES, ADM yields improved results compared to a simulation without subgrid model in the particle equation of motion. In the coarse LES the prediction of the filtered fluid turbulence kinetic energy is too high and since ADM adds the kinetic energy lost in the filtering, ADM only makes the results worse. They also considered a subgrid model based on fractal interpolation, but this did not give any improvement of the results. The same authors studied the effects of different filter widths and Stokes numbers in an *a priori* and *a posteriori* study of the same flow [63]. Although for coarser LES resolutions the filtered fluid velocity fluctuations are over-predicted, the particle wall accumulation and local segregation always appear to be under-predicted.

Gobert [42] presented an analytical method to assess the various subgrid models for the particle equation of motion, focussing on ADM [51] and two stochastic models [91, 94]. His conclusion was that the stochastic models are able to predict first and second moments of particle velocity accurately for smaller values of the Stokes number, whereas ADM performs better at higher Stokes numbers. For homogeneous isotropic turbulence Gobert and Manhart [43] proposed a special way of interpolation to retrieve the correct spectral properties of the fluid velocity at the particle position. In this way they obtained better results for statistical properties of the particles than with ADM, both for velocity fluctuations and for preferential clustering. A prerequisite of this model is that a model spectrum should be available, which is not as easy for inhomogeneous turbulent flows.

Bianco et al. [13] quantified the filtering error in LES of particle-laden turbulent channel flow by means of an *a priori* study. They considered filters of different type and width and particles of various Stokes numbers. In this way they determined the statistical properties of the filtering error as a function of the distance to the wall, which can be used to find the requirements a subgrid model in the particle equation of motion should satisfy. This research was extended by Geurts and Kuerten [41] by considering channel flow at Re_*τ*_ ranging between 150 and 950 and for particles of various Stokes numbers. They considered the use of a combination of ADM and a stochastic model and determined the properties of the stochastic part of the model by means of an *a priori* study. A striking result is that the RMS of the stochastic contribution to the fluid velocity is independent of Reynolds number and Stokes number if considered at the same wall-normal position in wall units.

Michałek et al. [70] used this result to develop a hybrid stochastic-deconvolution model for LES of particle-laden channel flow. In order to satisfy the well-mixed condition, *i.e*, the property that passive tracers stay uniformly distributed, an additional term in the Langevin equation for the stochastic part of the fluid velocity turned out to be required, which is a function of the Stokes number. The resulting model showed better results for Re_*τ*_=950 than simulations based on ADM alone.

All subgrid models developed for LES of particle-laden flow have in common that the Stokes number has a significant effect on the quality of the model.

### LES of one-way and two-way coupled particle-laden flow

Since 2005 quite some research has been devoted to subgrid modeling of the effects of the unresolved scales in the fluid velocity on the motion of particles in LES of particle-laden turbulent flow with one-way coupling. Unfortunately, similar efforts have not been made yet for LES with two-way coupling. It is known from DNS that particles may have an appreciable influence on the turbulence kinetic energy and this influence occurs mainly in the scales that are unresolved in an LES. The effects of this on the resolved scales should be taken into account in the two-way coupling force in LES of particle-laden flow.

Probably the first point-particle LES has been performed by Deardorff and Peskin [29] for homogeneous shear flow. They do not mention the term large-eddy simulation at all in their paper, but they did consider the spatially averaged Navier-Stokes equation for the fluid and used a spatially end temporally variable small-scale eddy coefficient to model the effects of the subgrid scales. They tracked 48 passive particles in the flow. Effects of the subgrid scales on the particle motion were not taken into account. They calculated particle dispersion, velocity autocorrelation coefficients and statistical properties of particle separation. Uijttewaal and Oliemans [108] performed point-particle DNS and LES to investigate particle dispersion and deposition in turbulent pipe flow at various Reynolds numbers. The particle equation of motion included only drag and lift forces and buoyancy. Due to the low particle volume fraction two-way coupling and particle collisions have not been applied here. Moreover, effects of subgrid scales of the fluid velocity have not been taken into account. It appeared that the particle relaxation time plays an important role in the deposition behavior of the particles.

Wang and Squires [115] studied the deposition of particles in vertical turbulent channel flow by means of large-eddy simulation for particle volume fractions so low, that the effects of the particles on the turbulence and the effects of particle collisions are negligible. They used the dynamic eddy-viscosity model as a subgrid model in the fluid equations [39]. They included the drag and lift force exerted by the fluid on the particles, but only the lift force due to the mean shear, which is in the wall-normal direction. They compared particle deposition rates with DNS results [69] and found good agreement. They also studied the effects of the subgrid scale velocities on particle deposition by solving an additional transport equation for the subgrid scale turbulence kinetic energy and observed only a small effect.

Boivin et al. [17] performed LES of gas-solid flows in forced homogeneous isotropic turbulence with two-way coupling. They applied several subgrid models in the fluid equations and several LES resolutions in both *a priori* and *a posteriori* tests. An increase of the particle mass loading results in a decrease in the dynamic constant in the dynamic eddy-viscosity model. An important conclusion of this paper is that at larger mass loadings a large part of the total dissipation in the flow is a result of interaction between fluid and particles. Therefore, the authors stipulate that modeling errors could have a smaller impact than in single-phase flows.

DNS results of a temporal mixing layer with evaporating droplets have been used to assess models for large-eddy simulation by Okong’o and Bellan [76]. Various options for the fluid properties at the droplet locations are considered: the real fluid property, which is available in a DNS, the filtered value, a random model based on a Gaussian distribution with known mean value and standard deviation and a deterministic model based on approximate deconvolution. It was observed that the deterministic model gives in general the best agreement with the DNS results, while the random model performs worse than the simulations without model for the subgrid term. In a later paper [55] *a posteriori* tests of the same problem have been performed. Here, however, no subgrid model was applied in the equations for the droplets, but the filtered fluid quantities were used. In this LES computational droplets were used, where each computational drop represents a fixed number of physical drops. In this way the number of droplets tracked in the simulations could be reduced by a factor of 32 as compared to the DNS without reducing the accuracy, provided that accurate subgrid models are applied in the fluid equations.

### LES of four-way coupled particle-laden flow

One of the first papers in which four-way coupling is applied in point-particle LES is by Yamamoto et al. [117] for flow in a vertical channel at Re_*τ*_=644. They considered particle volume fractions up to 1.4×10^−4^ and studied the influence of particle collisions. They included the drag force, the lift force due to particle rotation and due to shear and the gravity force on the particles, and they applied a deterministic particle collision model. They showed that particle collisions have a large effect on particle concentration and velocity statistics, even at the low volume fraction studied in this paper. This result is in agreement with later results where DNS is applied instead of LES [54].

A higher particle volume fraction of 0.013 has been studied by Vreman et al. [112] in LES of turbulent channel flow with four-way coupling. In this work only drag and gravity have been taken into account. The particles have a large effect on the turbulence. They result in a thinner boundary layer, increased gas velocity fluctuations in the streamwise direction and decreased in the other two directions.

Malloupas and Van Wachem [60] performed LES of particle-laden channel flow at a particle volume fraction of 4.8×10^−4^. They considered the effects of two-way and four-way coupling separately, compared the hard-sphere and soft-sphere collision models and studied the effects of various subgrid models in the LES. Moreover, they considered both smooth and rough walls. They compared their results with experimental data for a bulk Reynolds number of 42,000 based on the height of the channel. They observed that the differences between the Smagorinsky subgrid model [95] and the dynamic model [39] are very similar due to the relatively high resolution of the simulation. Particle collisions and the rough-wall model have a large effect on the results and lead to better agreement with experimental data. The difference between results of the hard-sphere and soft-sphere collision models is small.

Breuer and Aletto [18] performed point-particle LES with four-way coupling, using an efficient deterministic collision search algorithm. They used the model of Pozorski and Apte [80] as a subgrid model in the particle equation of motion. They investigated the effects of the particles and of the collisions in particle-laden flow in a turbulent channel at a bulk Reynolds number of 11,900, based on half the channel height and for a particle volume fraction of 7.3×10^−5^. For this volume fraction the effect of the particles on the turbulence is very small, but the effect of particle collisions is large. This is in agreement with observations found for point-particle DNS of turbulent channel flow at a similar particle volume fraction [54]. The results of this study are in good agrement with experimental results. In the same paper a second application is investigated of cold flow in a combustion chamber, where a jet of particle-laden flow mixes with an unladen annular flow. Two different mass loadings have been considered. For the highest mass loading of 110 % the particles have an appreciable influence on the turbulence. Also for this application good agreement with experimental results was obtained.

In a later paper [2] the same authors studied four-way coupled point-particle LES of horizontal pipe flow at a bulk Reynolds number of 120,000 based on the diameter op the pipe. They considered poly-disperse particles and took into account particle rotation and apart from the drag and buoyancy forces also the Saffman lift force and the Magnus force due to particle rotation. They compared their results with experimental results in a smooth glass pipe and in a rough steel pipe, where the roughness is modeled in the simulations by using a wall-normal and tangential restitution coefficient unequal to 1, resulting in momentum loss and by considering the shadow effect, which becomes important for smaller impact angles. They especially focused on secondary flow, which appeared not te be driven by the particles.

Breuer and Almohammed [19] included a model for particle agglomeration to four-way coupled point-particle LES of turbulent channel flow. They added a cohesive Van der Waals force between two colliding particles, which makes it possible that particles stick together after the collision and move with the same velocity. They implemented several models for the particle after agglomeration, all assuming a spherical shape. In one model the volume of the particles is conserved and the mass density constant, in a second model the moment of inertia around the center of mass is conserved, and in the third a closely-packed sphere is assumed. In the latter two models the mass density of the agglomerate is smaller than that of the original particles. The final results of the three models are only slightly different. The authors studied collision and agglomeration rates as functions of the wall-normal coordinate and investigated the effects of two-way coupling, the use of a subgrid model in the particle equation of motion and the use of lift force. These three together result in a significant reduction in the rates of both collision and agglomeration.

## Perspective

The latest three decades have shown a rapidly growing number of research papers on numerical simulation of particle-laden flows. The aim of these papers has been both the increase of our fundamental understanding of these flows and the solution of industrial problems. Regarding Lagrangian methods, the most common methods are still point-particle DNS and LES. However, these approaches require knowledge of the forces exerted by the surrounding fluid on a particle. For smaller particles expressions for these forces are well known, but some inherent problems remain. The most important of them are that they are based on the undisturbed fluid velocity at the location of the particle, which is not well-defined and not accessible in experiments, and the way the reaction forces exerted by the particles on the fluid should be treated. For larger particles, accurate expressions for the forces are not well known, and it is uncertain whether a point-particle approach can be applied in this case at all.

Solutions for both problems can be obtained from particle-resolved simulations, in which the flow around each particle is solved in detail. Particle-resolved simulations are still only possible for relatively small numbers of particles, that are not too small, but recent years have witnessed an enormous increase in the capabilities of these methods. Recently, particle-resolved simulations with 10,000 particles in turbulent channel flow have been performed by means of the immersed boundary method [78]. Also, progress can be seen in the development of numerical methods for particle-resolved simulations with body-fitted grids [111]. As has recently been observed by Prosperetti [82], this progress marks the beginning of a golden age of science in the area of particulate flows, or more general, multiphase flows.

Particle-resolved direct numerical simulation has the capability to study the force on a particle in turbulent flow and gives direction on the way this force can be modeled in point-particle direct numerical simulations, preferably without the use of the undisturbed fluid velocity. Also, the way in which the two-way coupling force should be applied in point-particle simulations can be found from particle-resolved simulations. An example of this can be found in the recent paper on particle-resolved DNS of the flow around a fixed array of particles in homogeneous isotropic turbulence by Vreman [111]. There it was found that the agreement with point-particle DNS can substantially be improved by distributing the two-way coupling force in the point-particle DNS over a larger volume than usually chosen. This systematic study into the two-way coupling force and the way it should be distributed in space, takes away some of the fundamental objections against two-way coupling in point-particle DNS, as expressed by Eaton [32].

Due to the enormous gap in the number of particles that can be handled in particle-resolved DNS and industrial or environmental applications, point-particle methods will necessarily play a large role for many years to come, but the combination of the two techniques can force a breakthrough in the predictive capabilities of point-particle DNS, which could turn this method into a viable tool in many applications.

However, DNS is still a very time-consuming method for real applications. Even for single-phase flows it can be expected that RANS and LES will remain prominent during the forthcoming years. Therefore, there is still a need for progress in particle-laden LES and RANS, and also in Euler-Euler simulation methods. Whereas quite a lot of effort has been spent onto subgrid modeling in one-way coupled LES of particle-laden flow, and in particular on the modeling of the subgrid contribution to the fluid velocity in the drag force, substantially less research has been performed on two-way coupling in point-particle LES and on other forces exerted by the fluid on a particle. Since the particles usually change the energy spectrum of the fluid in the unresolved scales in LES, it is not a priori clear whether and how this two-way coupling can be modeled. A systematic study of (filtered) results of particle-laden DNS can provide insight into this, in the same way as has been done for one-way coupled simulations. Although in the meantime LES of particle-laden flows has been performed for several applications with more and more complex physics, such as chemical reactions, phase change and agglomeration, a better fundamental understanding of two-way coupling in LES is required for this field of research to progress.
